# Spatial Differentiation and Tradeoff–Synergy of Rural Multifunction at the County Scale in Anhui Province in the China’s Traditional Agricultural Areas

**DOI:** 10.3390/ijerph192013604

**Published:** 2022-10-20

**Authors:** Rongtian Zhang

**Affiliations:** Institute of Rural Revitalization Strategy, Yangzhou University, Yangzhou 225009, China; rtzhang@yzu.edu.cn

**Keywords:** rural multifunction, spatial differentiation, tradeoff, synergy, county scale, Anhui Province, traditional agricultural areas

## Abstract

The study of rural multifunction interaction has ignored the spatial differentiation characteristics, so it is necessary to reveal in-depth the spatial interaction relationship of rural multifunction interaction on the basis of type division and pattern recognition at the county scale. Taking Anhui as a case study, based on the county scale, the paper constructed an index system of rural multifunction evaluation, and comprehensively applied the improved entropy method, spatial autocorrelation model, and Spearman correlation coefficient to study the temporal-spatial characteristics and tradeoff–synergy relationship of rural multifunction from 2000 to 2020. The results showed that (1) the overall rural production function at the county level in Anhui showed an upward trend, showing a spatial pattern of “low in the south and high in the north”. The rural life function was gradually weakened, showing the characteristics of gradual decline from suburban to exurb. The rural ecological function increased, showing a high value distribution in areas with rich mountains and hills or a dense water network. (2) The spatial concentration of HH (High-High) and LL (Low-Low) positive correlation types of rural production, life, and ecological function was significant during the study period. The negative correlation types of HL (High-Low) and LH (Low-High) had low spatial agglomeration and showed discrete distribution. (3) There was a spatial synergistic relationship between rural production and life function at the county scale in Anhui Province. The tradeoff–synergy relationship between rural production and ecological function and between rural life and ecological function showed a trend of synergistic–tradeoff fluctuation. The spatial difference of the rural multifunction tradeoff–synergy relationship was significant during the study period. (4) In the future, we should change the situation that the expansion of rural production function compresses rural life and ecological function, and promote rural life and ecological function to promote the benign and coordinated development of rural multifunction at the county scale in traditional agricultural areas.

## 1. Introduction

Rural is a concept of a spatial region system, which refers to the region outside the city. With rapid urbanization and the development of industrialization, the relationship between urban and rural areas is also changing, radiating and driving the role of cities to the countryside to strengthen gradually. The village in the urban and rural regional system functions presents a trend of diversification; the rural regional system is gradually moving from the traditional single function of agricultural production to industrial, leisure services, retail and other multifunction shift, and the characteristics of the evolution of rural regional functions from single to diversified and complex are increasingly prominent [1,2]. Meanwhile, in the national Rural Revitalization Strategic Plan, it was clearly proposed to give play to the multiple functions and values of rural areas [3,4]. Against the background of a rural revitalization strategy, the scientific evaluation of the level of regional rural multifunction, revealing the regional differentiation and spatial relationship of rural multifunction, and analyzing the driving mechanism of their formation and evolution have become an important practical proposition for promoting the rural revitalization development.

Multifunctionality originated from the agricultural sector. Japan first proposed the concept of agricultural functionality to protect rice culture [5]. After the 1980s, the idea of multifunctional agriculture appeared in relevant policy documents. Agenda 21, issued by the Rio Earth Summit (1992), officially proposed that agricultural policies should consider the multifunctional characteristics of agriculture for the first time [6,7]. At the end of the 20th century, the 2000 Agenda proposed the creation of a “European agricultural model” to protect agricultural pluripotency and its role in the economy, environment, and society. With the introduction of the 2000 Agenda, European rural development policy began to practice multifunction, and the EU took “versatility” as the guide for rural construction in the anti-urbanization stage [8]. The theory of rural multifunction originated from the theory of multifunction agriculture put forward at the end of the 20th century. The theory of rural multifunction is a new theoretical perspective put forward by Western developed countries after their rural development experienced the paradigm of productivism and post-productivism, and it is a further deepening of the theory of multifunction agriculture [9,10]. As an essential feature of rural areas, multifunctionality contains the multiple goals of rural development and the multiple demands of economic and social development on rural areas. It has become the core force of rural reform and a theoretical perspective on the law of rural development [11]. A survey of the literature showed the progression of rural multifunction research as follows. (1) The research content was increasingly rich, mainly including connotation characteristics of rural multifunction [12,13], the evaluation and zoning of rural multifunction [14,15,16], spatial-temporal changes of rural multifunction [17,18,19], the relationship between rural multifunction and rural transformation [20], and revitalization path research from the perspective of multifunctional [21]. (2) The scale of research has been continuously deepened, mainly involving the macroscopic scale (urban agglomeration [22], metropolitan area [23]), meso-scale (city [24], county [25]), and the microscopic scale of the village area [26]. However, multi-scale comprehensive comparative studies are relatively few in number. (3) Comprehensive integration of research methods, mainly involving quantitative analysis models such as comprehensive evaluation method [27], Dagum coefficient [28], SOFM model [29], and semi-variometric function method [30], and the GIS spatial analysis method began to be applied to rural multifunction analysis [31]. (4) At present, rural multifunction research has focused more on the quantitative relationship and relatively neglects the characteristics of spatial differentiation [32,33]. Meanwhile, research on the evolution characteristics and mechanisms of rural multifunction in different counties has been relatively weak, and it is also necessary to further explore the level, temporal evolution, and spatial interaction of rural multifunction structure on the basis of type division, zoning evaluation, and pattern identification at the county scale.

The concept of the tradeoff–synergy relationship was first derived from the research on ecosystem services. The complex tradeoff relationship among different ecosystem services is called the tradeoff relationship, and the mutual gain relationship is called the synergy relationship [34,35]. At present, the study of the tradeoff–synergy relationship has been applied to the research fields such as the multifunctional system of cultivated land resources [36], the functional system of land use [37], and the functional system of rural landscape [38], etc. These results have provided a reference for exploring the rural multifunction interaction relationship. The rural area is a multifunctional complex system of production, life, and ecology. The study on the tradeoff–synergy relationship of rural multifunction will help to enrich the theoretical results of rural regional ecosystem services, and also provide a new perspective for the research on the development of rural regional multifunction [39]. In light of this, the paper tried to introduce the research method of tradeoff–synergy ecology into the analysis of the rural multifunction relationship. Anhui Province, a major grain-producing area in China, was selected as a typical case. Based on the county scale, the period from 2000 to 2020 was taken as the study period. The improved entropy method was used to quantitatively evaluate the rural multifunction level in Anhui. The spatial autocorrelation model and Spearman correlation coefficient were used to study the spatial-temporal differentiation and tradeoff–synergy relationship of rural multifunction in Anhui. On this basis, the paper put forward some suggestions for the coordinated development of rural regional functions in Anhui, in order to provide practical reference for the optimization and coordinated development of rural regional functions in the main grain producing areas in China. The structure of the manuscript mainly includes there parts, as follows:

- How can we scientifically measure the level of rural multifunction at the county scale? In this part, based on the interpretation of the connotation of rural multifunction, the paper constructed the evaluation index system of rural multifunction from the dimensions of production, life and ecology, and used the improved entropy method to measure the level of rural multifunction at the county scale in Anhui in China’s traditional agricultural areas.
- How can we reveal the characteristics of rural multifunction spatial differentiation at the county scale? In this part, the paper focused on the characteristics of the overall pattern, functional spatial differentiation, and spatial correlation pattern to reveal the spatial-temporal variation of rural multifunction patterns at the county scale in Anhui in China’s traditional agricultural areas.
- How can we reveal the tradeoff–synergy of rural multifunction at the county scale? In this part, the paper mainly used the Spearman correlation coefficient, bivariate LISA value, and other methods to reveal the temporal characteristics and spatial differentiation of the rural multifunction tradeoff–synergy relationship at the county scale in Anhui in China’s traditional agricultural areas.

## 2. Materials and Methods

### 2.1. The Study Area

**Figure 1:**
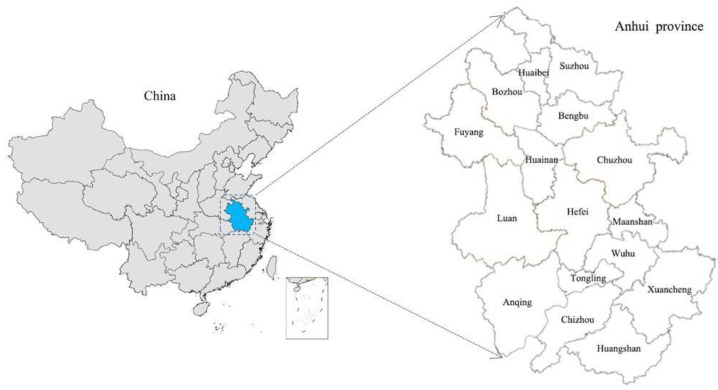
The geographical location of the study area. Source: Based on the standard map service website of the Ministry of Natural Resources with a scale of 1:48 million.

Anhui Province is located in the central region of China (Figure 1), with a total land area of about 139,600 square kilometers, accounting for 1.45% of the total land area of China. Anhui is an important agricultural production, raw materials, and processing and manufacturing base in China, and agricultural product processing occupies an important position in the country. By the end of 2020, Anhui’s GDP reached CNY 3868.063 billion, with per capita GDP of only CNY 63,426. The total output value of agriculture, forestry, animal husbandry, and fishery is CNY 56.8 million. In the process of rapid urbanization and industrialization, Anhui’s rural regional elements, structure, and function have shown accelerated transformation, which represents an important epitome of China’s rural regional function transformation after the reform and opening up. Therefore, the selection of Anhui Province as a typical case study of China’s traditional agricultural area is representative to some extent.

### 2.2. Research Methods

#### 2.2.1. Improved Entropy Method

At present, according to the different ways of determining weights, the comprehensive evaluation methods of indicators can be roughly divided into two categories, namely, the subjective weighting evaluation method (analytic hierarchy process, expert scoring method) and the objective weighting evaluation method [40]. Among them, the entropy method is an objective weighting evaluation method, which can effectively overcome the subjectivity of artificially determining the weights and the overlap of information among multiple indicators [41,42]. This method is suitable for multi-index quantitative evaluation, and has been applied in different fields such as urbanization comprehensive evaluation, tourism competitiveness evaluation, and cultivated land ecological security evaluation [43]. Meanwhile, in order to overcome the disadvantages of extreme value or negative value in the measurement results of traditional entropy method, the index data are processed by using standardized transformation method (i.e., the improved entropy method). See [44] for the theoretical calculation steps.

#### 2.2.2. Spatial Autocorrelation Model

The spatial autocorrelation model describes and visualizes the spatial distribution pattern of geographical phenomena, and discovers the spatial clustering characteristics and analyzes the mechanism. The specific indicators of the model include Global Moran’s I and Local Moran’s I index [45,46], and the theoretical formula is as follows:

① Global Moran’s I

$$I\left(d\right)=\frac{\sum_{i=1}^{n}\sum_{j=1}^{n}\left(X_{i}-\bar{X}\right)\left(X_{j}-\bar{X}\right)}{S^{2}\sum_{i=1}^{n}\sum_{j=1}^{n}W_{ij}}$$

where $X_{i\;}$ is the observed value of the region *i*; $X_{j\;}$ is the observed value of the region *j*; $W_{ij\;}$ is the spatial weight matrix, and the spatial adjacency is 1. I(d)>0 is a positive spatial correlation, and there is significant agglomeration in space.

② Local Moran’s I

$$I_{i}=\frac{Z_{i}-\bar{Z}}{D^{2}}\overset{n}{\underset{j=1}{\sum }}W_{ij}\left(Z_{j}-\bar{Z}\right)$$

where $Z_{i}^{\text{'}}$ and $Z_{j}^{\text{'}}\;$ are the standardization of the observed values on regional *i* and *j*. When $I_{i}>0$, it indicates that the agricultural multifunction has a small difference with the surrounding areas. When $I_{i}<0$, it indicates that the agricultural multifunction is significantly different from the surrounding area.

#### 2.2.3. Spearman Correlation Coefficient

Spearman correlation coefficient is a non-parametric statistical method that uses the rank size of two variables to perform linear correlation analysis. This method has been applied to ecosystem service tradeoff–synergy research [47,48]. In light of this, the Spearman correlation coefficient method was used to quantitatively analyze the tradeoff–synergy of rural multifunction at the county level. Its basic principle is as follows: the $\left\{\left(X_{i},Y_{i}\right)\right\}$ denotes *n* pairs of independent identically distributed data, since the childhood of the $X_{i}$ arrangement can obtain a new set of data: $X_{\left(1\right)}<X_{\left(2\right)}<X_{\left(3\right)}\cdots <X_{\left(n\right)}$. The $Y_{i}$ corresponding to the order statistic of *x* is called the adjoint of $X_{i}$ [49], and the formula is as follows:

$$r_{s}\left(X_{i},Y_{i}\right)=1-\frac{6\sum_{1}^{n}{\left(P_{i}-Q_{i}\right)}^{2}}{n\left(n^{2}-1\right)}$$

where $P_{i}$ refers to $X_{i}$ is the *k* position in the sequence $\left\{\left(X_{i}\right)\right\}$; *k* is the rank of $X_{i}$; similarly; $Q_{i}$ is the rank of $Y_{i}$. If the correlation is positive and significant, it indicates that there is a synergistic relationship between the two rural regional functions. If the correlation is negative and significant, it indicates that there is a tradeoff between the two rural regional functions.

### 2.3. Indicator System

Rural areas have multifunctional properties, which not only provide food security for rural residents, but also provide housing security for rural people. Moreover, they are also an important carrier to maintain regional ecological security, with multiple functions such as production, life, and ecology [50]. Therefore, the connotation of rural multifunction refers to the sum of various functions provided by rural areas to meet the various needs of rural residents in a certain social development stage. It not only emphasizes the comprehensive role of rural areas in production, life, ecology, and other aspects, but also emphasizes the interaction and cooperation effect of various functions within the multifunctional system [51]. Throughout the relevant studies, it can be found that most of the current studies were based on the regional characteristics of the rural system and mainly built their rural multifunction evaluation index system from different dimensions of production, life, and ecology [52,53]. In light of this, on the basis of understanding the connotation characteristics of rural multifunction, and considering the actual situation of the Anhui county rural areas, 15 specific indicators were selected from the three dimensions of production, life, and ecological function to construct the theoretical system of county rural multifunction evaluation (Table 1).

(1) Production function dimension. The country is mainly engaged in agricultural and non-agricultural production areas, which is the most basic function of rural areas. The indicators of per capita grain output (*X*_1_), crop output per unit area (*X*_2_), agricultural output value per unit area (*X*_3_), proportion of total output value of agriculture, forestry, animal husbandry and fishery (*X*_4_), and non-agricultural employment rate of rural employees (*X*_5_) were mainly selected. (2) Life function dimension. Rural areas are the main areas where the rural population is concentrated and lives, providing living functions for rural residents. The Engel coefficient (*X*_6_), rural per capita electricity consumption (*X*_7_), number of beds per 10,000 people (*X*_8_), rural per capita net income (*X*_9_), and urban–rural income ratio (*X*_10_) were mainly selected. (3) Ecological function dimension. The countryside is an important carrier of providing ecological products and suitable living environment for urban and rural residents. The average ecological service value (*X*_11_), forest coverage rate (*X*_12_), average pesticide use (*X*_13_), average fertilizer use (*X*_14_), and average film use (*X*_15_) were mainly selected.

### 2.4. Data Collection

The research data of the paper mainly came from two parts: ① Economic and social data. These mainly came from the Anhui Statistical Yearbook (2001–2021), Anhui Agricultural Economic Statistical Yearbook (2001–2021), and China County Statistical Yearbook (County-City Volume) (2001–2021). ② Spatial data. The research scale of this paper was county space including nine county-level cities, 50 counties, and 16 municipal districts, with a total of 75 county units, which were mainly derived from the Administrative Zoning Map of Anhui Province, which was obtained by vectorization in ArcGIS10.2 analysis software, and the adjusted administrative units were processed.

## 3. Results

### 3.1. Rural Multifunction Spatial Differentiation

#### 3.1.1. Characteristics of Overall Pattern Change

**Figure 2:**
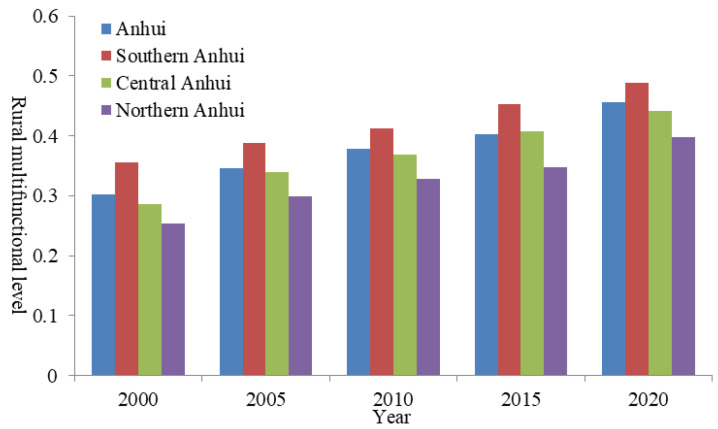
Rural multifunction level and regional differences in Anhui from 2000 to 2020.

The improved entropy method model was used to calculate the rural multifunction index in Anhui from 2000 to 2020 (Figure 2). The results showed that since 2000, the rural multifunction level in Anhui has shown a continuous improvement trend from 0.3023 in 2000 to 0.3786 in 2010, and then to 0.4553 in 2020. The rural multifunction level at the county scale in Anhui has increased by 50.61% in the past 20 years. From the perspective of different regions, the rural multifunction level in Southern Anhui (Maanshan, Wuhu, Tongling, Chizhou, Huangshan, Xuancheng) fluctuated in the interval of [0.3542, 0.4889] from 2000 to 2020, while the rural multifunction level in Central Anhui (Hefei, Chuzhou, Luan, Anqing) fluctuated in the interval of [0.2863, 0.4408]. However, the rural multifunction level in Northern Anhui (Suzhou, Huaibei, Bozhou, Fuyang, Bengbu, Huainan) fluctuated in the range of [0.2539, 0.3983]. Therefore, the overall regional difference of rural multifunction level at the county scale in Anhui during the study period was as follows: Southern Anhui > Central Anhui > Northern Anhui, and the rural multifunction level showed a basic regional difference characteristics of “high in the south and low in the north”.

#### 3.1.2. Characteristics of Functional Spatial Differentiation

**Figure 3:**
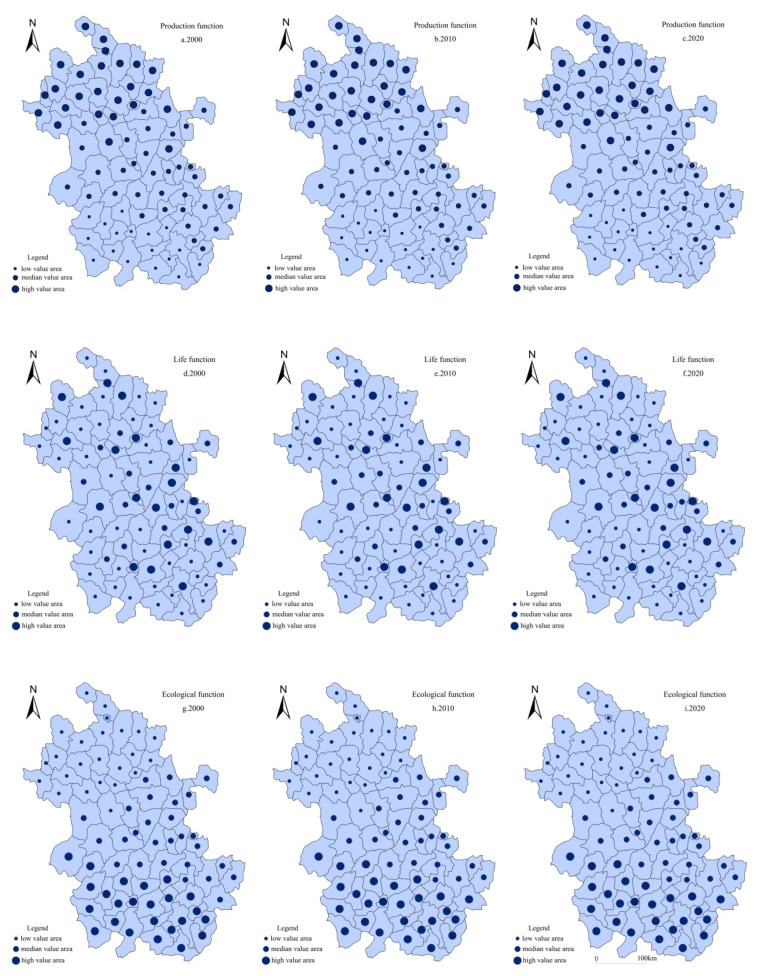
The spatial differentiation of rural production, life, and ecological function in Anhui.

Based on ArcGIS10.2 software and the Jenks breaking point method, the rural production, life, and ecological function were divided into three types: high value area, median value area, and low value area. The spatial-temporal pattern change characteristics of rural multifunction in Anhui from 2000 to 2020 were mainly revealed from the three dimensions of production, life, and ecological function (Figure 3).

(1) Spatial differentiation of rural production functions at the county scale. From 2000 to 2020, the spatial pattern of rural production function at the county scale in Anhui showed a trend of “low in the south and high in the north”. In 2000, the high value of rural productive function in Anhui was mainly distributed in Fuyang, Suzhou, Huaibei, and other counties in the north of Anhui, while the low value was mainly clustered in Huangshan, Anqing, Chizhou, and other counties in the southwest of Anhui. In 2010, only some counties such as Yingshang County had spatial replacement. By 2020, the high value of rural production function in Anhui was still concentrated in Northern Anhui, which was mainly attributed to the flat terrain and relatively superior agricultural production conditions in Northern Anhui; this was an important grain production area in Central China, forming a relatively concentrated area of rural production functions.
(2) Spatial differentiation of rural life functions at the county scale. The spatial distribution of rural life functions in Anhui showed a decreasing pattern from the inner suburbs to the outer suburbs. Among them, the high value areas of rural life function in 2000 were mainly distributed in Hefei, Wuhu, Huainan, and other urban districts and suburban counties, while the low value areas were mainly clustered in Linquan, Susong, Jinzhai, and other outer suburban counties. In 2020, counties with a high value of rural life function were still mainly clustered in urban districts and suburban counties, and the spatial distribution of the high value of production function was stable. This was mainly attributed to the relatively high level of urbanization development in urban districts and suburban counties, which were greatly influenced by the urbanization radiation, complete rural infrastructure, and public service facilities as well as the relatively high level of regional rural life functions.
(3) Spatial differentiation of rural ecological functions at the county scale. The spatial pattern of rural ecological functions in Anhui from 2000 to 2020 showed the characteristics of high value distribution along mountains and rivers. Among them, the higher value of rural ecological function in 2000 was mainly distributed in the counties of Luan, Chizhou, and Huangshan, and Wuhu and Maanshan counties. By 2020, only a few counties (Ningguo, Shucheng) have made minor adjustments, and the high-value rural ecological function areas were still mainly concentrated in the Southwest Anhui and Wangjiang counties. This was mainly due to the fact that these counties are close to mountains and rivers, the county ecological environment quality is good, and the county spatial proximity effect has formed high-value rural ecological function agglomeration areas in Anhui since 2000.

### 3.2. Rural Multifunction Spatial Correlation Pattern

In order to explore the relationship between rural production, life, and ecological functions in Anhui during the study period, the spatial autocorrelation model was used to identify the spatial correlation of each single rural function (Table 2). Table 2 shows that at the test level of 0.1%, the estimated Global Moran’s I values of rural production, life, and ecological function in 2000, 2010, and 2020 were all positive. The calculation results of the Global Moran’s I index showed that rural production, life, and ecological function in Anhui at the county scale had obvious positive spatial correlation. That is, the adjacent county units had strong interaction and spatial agglomeration. Among them, the Global Moran’s I value of rural production function level in Anhui during the study period was between [0.4532, 0.5036], the Global Moran’s I value of rural life function level was between [0.3015, 0.3452], and the Global Moran’s I value of rural ecological function level was between [0.3672, 0.4038], which indicated that the agglomeration characteristics of rural life function in Anhui were the most significant, while the agglomeration characteristics of rural production and ecological function were relatively weak.

**Figure 4:**
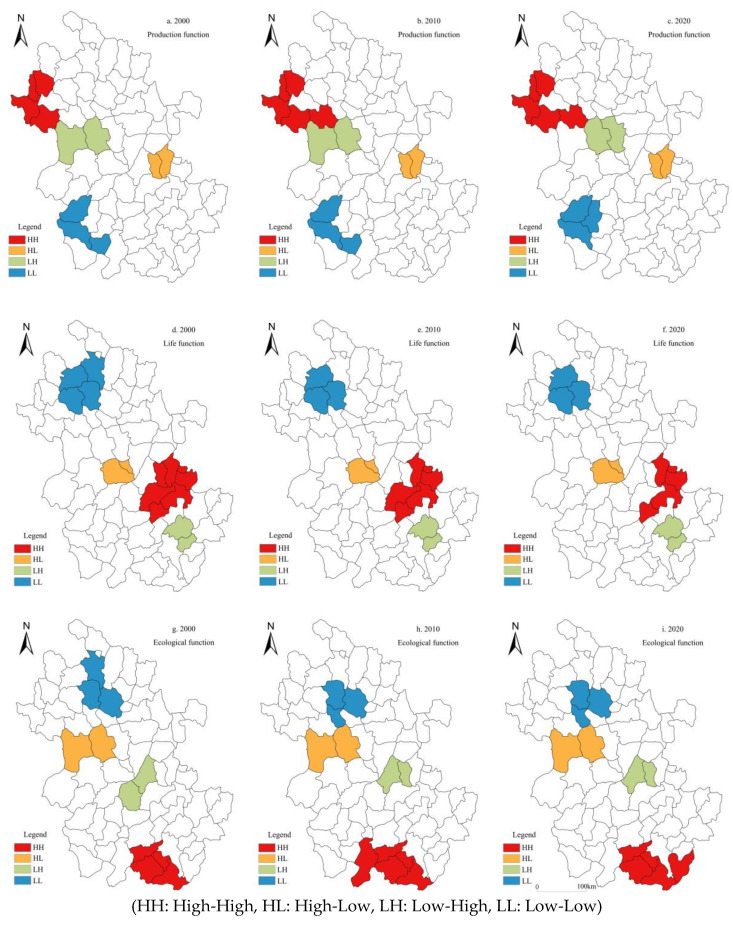
Cluster map of the rural production–life-ecological function at the county scale in Anhui.

Based on the global spatial autocorrelation, a LISA cluster map was used for local spatial autocorrelation analysis to further explore the local spatial heterogeneity of rural multifunction in Anhui. The results showed that the spatial heterogeneity of rural production, life, and ecological function in Anhui was significant from 2000 to 2020. (1) Rural production function: There was little change across the whole 20 years. The number of HH county units increased from 4 to 5, and HH counties were spatially clustered in Linquan, Jieshou, Funan, and other counties of Fuyang. The LL type was mainly concentrated in Western Anhui such as Yuexi, Taihu, Wangjiang, and other counties. The number of HL and LH counties did not change, and only the spatial agglomeration types of Huoqiu and Changfeng counties were fine-tuned (Figure 4a–c). (2) Rural life function: During the study period, the number of HH county units decreased from 7 to 5, and the HH agglomeration area was reduced, mainly concentrated in Wuhu–Maanshan–Tongling area. The number of LL type counties did not change significantly, and were mainly concentrated in Suixi, Woyang, Mengcheng, and other counties in Northern Anhui. The number of HL and LH type counties was relatively small, showing a decentralized spatial layout (Figure 4e,f). (3) Rural ecological function: In 2000, 2010, and 2020, the number of HH counties of rural ecological function in Anhui increased slightly, mainly concentrated in Qimen, Yixian, and Xiuning in the mountainous area of Southern Anhui. LL agglomeration was mainly distributed in Suixi and Fengtai Counties in Northern Anhui. The number of HL and LH type county units was small, mainly scattered in Lu ‘an, Hefei and other counties (Figure 4g–i). In general, the spatial agglomeration characteristics of the positive correlation types (HH, LL) of rural production, life and ecological function in Anhui from 2000 to 2020 were significant, showing the distribution pattern of a “cluster”. However, the negative correlation types (HL, LH) had low spatial agglomeration and showed “scattered” distribution characteristics.

### 3.3. Rural Multifunction Tradeoff–Synergy Relationship

In order to characterize the interaction between rural multifunction from 2000 to 2020, based on SPSS18.0 software (International Business Machines Corporation, Armonk, USA), with the help of R language (Ross Ihaka, Auckland, New Zealand) and the corrgram package, the Spearman correlation coefficient was used to realize the calculation of the rural multifunction tradeoff–synergy relationship in Anhui from 2000 to 2020, and the spatial differentiation of rural multifunction tradeoff–synergy was revealed through the bivariate LISA value.

#### 3.3.1. General Characteristics of Tradeoff and Synergy

According to the Spearman correlation coefficient method, the rural multifunction tradeoff–synergy relationship was judged. The positive correlation showed the mutual gain and orderly development of the coordination relationship, while the negative correlation showed the tradeoff relationship of one tradeoff and another tradeoff, disorder and chaos [50]. The absolute value indicated the degree of correlation, that is, the strength of the tradeoff–synergy relationship (Table 3).

(1) The rural production and life function showed a positive correlation from 2000 to 2020, indicating a spatial synergy. With the passage of time, the correlation coefficient increased from 0.3235 in 2000 to 0.5683 in 2020, and the degree of synergy between rural production and life function in Anhui increased. This was mainly attributed to the diversity of the farmers’ livelihood, which led to the continuous improvement in the farmers’ living standards, promoted the improvement of rural production and life functions, and the production life functions complemented each other.
(2) The tradeoff and synergy between rural production and ecological function showed a fluctuating trend from 2000 to 2020, with the correlation coefficient ranging from −0.1566 in 2000 to 0.2821 in 2010 and then to −0.2235 in 2020. This indicates that the rural production and ecological function in Anhui has changed from a weak synergy relationship to a strong tradeoff relationship. This is mainly because the rural development only pursued economic benefits and neglected the ecological environment, which affected the coordinated development of rural production and ecological function.
(3) The relationship between rural life–ecological function at the county level was characterized by weak tradeoff and then weak synergy. Specifically, the Spearman correlation coefficient was −0.3476 in 2000, 0.0966 in 2010, and 0.1276 in 2020. This trend was mainly attributed to the early rural domestic sewage, garbage, and other random dumping as the rural ecological environment had a negative effect; with the improvement in the rural living environment and the enhancement of the farmers’ awareness of environmental protection, it was helpful to promote the coordinated development of rural life–ecological function.

#### 3.3.2. Spatial Differentiation of Tradeoff-Synergy

In order to understand the spatial differentiation of the rural multifunction tradeoff –synergy relationship in Anhui Province during the study period, the rural multifunction bivariate Lisa values in 2000, 2010, and 2020 were calculated with the help of geoda095 software, and the spatial interaction relationship between the two rural functions in Anhui Province was expressed with the help of a LISA map.

**Figure 5:**
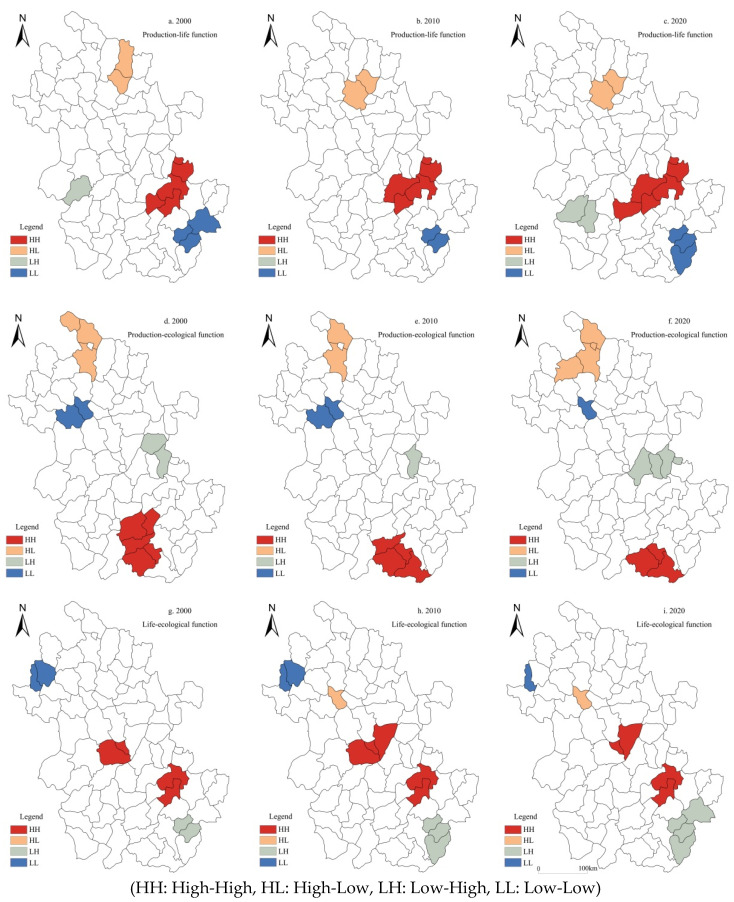
LISA expression of bivariate rural production, life, and ecological function in Anhui.

(1) Rural production–life function. In 2000, HH was clustered in Wanjiang urban agglomeration, LL was clustered in Jixi, Ningguo, and other counties in the south of Anhui. The tradeoff relationship was mainly distributed in the counties of Lingbi and Guzhen in Northern Anhui. In 2010, the synergy relationship of HH counties was mainly located in Wuhu–Maanshan–Tongling. The spatial distribution of tradeoff relationships (HL, LH) was less. By 2020, the spatial distribution of the tradeoff–synergy relationship was stable, and the synergy relationship was still mainly located in the Wanjiang River region (Figure 5a–c).
(2) Rural production–ecological function. In 2000, the HH type was clustered in Shitai, Dongzhi, and Qimen Counties in Southern Anhui. The LH type of tradeoff relationship was located in Quanjiao and Chaohu Counties. HL types were distributed in Fengtai, Yingshang, and other counties. In 2010, Xiuning evolved to the HH type, and Dangshan evolved to non-significant from the LL type. The spatial distribution of HL and LH types was scattered. By 2020, the synergy relationship was weakened, and the number of HH counties decreased. The tradeoff relationship was continuously enhanced, and the HL and LH types gradually evolved to Woyang, Hanshan, and Chaohu Counties (Figure 5d–f).
(3) Rural life–ecological function. In 2000, the HH type of synergy relationship was mainly distributed in the Hefei–Wuhu region; the LL type was concentrated in Jieshou, Taihe, and other counties in Northern Anhui. The tradeoff relationship mainly manifested as the LH agglomeration type. In 2010, the spatial patterns of the HH and LL agglomeration types of synergy relationship did not change greatly, and only Feidong evolved into the HH type. By 2020, synergy relationships were clustered in counties such as Feixi and Nanling, mainly of the HH type. The regional expansion of LH type was mainly distributed in Southern Anhui. HL type counties were mostly clustered in Huainan and other counties (Figure 5g–i).

## 4. Discussion

Rural multifunction is an objective attribute of rural areas. The development of rural multifunction conforms to the requirements of rural sustainable development, and is also an important paradigm of rural transformation and development in the future. The county is an important carrier to break the urban–rural dual structure and achieve urban–rural integration and development. Promoting multifunctional development at the county level is an important path to achieve urban–rural integration and rural revitalization under the new situation. In the context of the implementation of the current rural revitalization strategy, how to effectively explore the rural versatility is the meaning of the topic of rural revitalization and prosperity development [54,55,56,57,58]. In the future, we will combine the function status, resource characteristics, and regional background of different rural regional types in traditional agricultural areas, highlight the dominant functions of different regions, and adopt differentiated rural regional function optimization strategies according to local conditions:

(1) Function optimization of rural production advantage areas. In the future, the HH counties of rural production function in the north of Anhui should pay attention to the adjustment of planting structure to focus on grain production and carry out a variety of high value-added management methods such as planting flowers and seedlings and vegetables. We will develop the processing industry of agricultural products, livestock and poultry products, and the food manufacturing industry, extend the industrial chain, and promote the benign interactive relationship between industry leading agriculture and agriculture promoting industry.
(2) Function optimization of rural life advantage areas. Based on resource endowment and location advantage, the counties with a high value of rural life function should further strengthen rural infrastructure construction, improve rural basic public service function, deepen the adjustment and upgrade of county industrial structure, and promote the effective coordination of rural regional function.
(3) Function optimization of rural ecological advantage areas. In the future, the rural ecological function HH counties in Southern Anhui should strengthen the protection of rural ecological environment, prohibit the reclamation of sloping land and other unreasonable land resource utilization, and carry out comprehensive control of soil and water loss. We will take measures such as ecological compensation and fiscal transfer payments to internalize the external effects of regional ecological protection, create more ecological jobs, and continue to strengthen the function of regional rural ecological conservation. This paper puts forward some suggestions on the optimization of rural functions from the different dimensions of production–life–ecology, which are helpful in promoting the coordinated development of rural multifunction at the county scale in Anhui.

## 5. Conclusions

(1) The rural multifunction showed significant spatial differentiation in Anhui Province in China’s traditional agricultural areas. The evolution of rural multifunction in Anhui has shown a certain trend of improvement, showing the regional difference characteristics of Southern Anhui > Central Anhui > Northern Anhui. The rural production function showed an upward trend, showing a spatial pattern of “low in the south and high in the north”. The rural life function was weakened, showing a gradual decline from the suburbs to the outer suburbs. The rural ecological function was continuously enhanced, which showed the distribution pattern of high value agglomeration in the rich mountainous and hilly areas or the dense water network.
(2) The rural production, life, and ecological functions showed spatial autocorrelation characteristics, among which the rural life function aggregation was the most significant, while the production and ecological function aggregation was relatively weak. During the study period, the positive correlation types of rural production, life, and ecological function in Anhui were significantly clustered, showing a “cluster-like” pattern. However, the negative correlation types showed low spatial agglomeration and “discrete” distribution. This conclusion revealed the spatial distribution characteristics of the dominant function types of different rural areas in the traditional agricultural areas at the county scale, and revealed the functional pattern of rural areas at the county scale from the perspective of spatial relations.
(3) There was a spatial synergy relationship between the rural production–life functions and both the rural production–ecological functions and life–ecological functions showed a trend of tradeoff–synergy relationship evolution. With the help of the LISA map, it can be found that the spatial heterogeneity of rural production–life functions, production–ecological functions, and life–ecological functions of the tradeoff–synergy relationship was significant in Anhui from 2000 to 2020. This conclusion revealed the relationship between rural regional functions at the county scale, and provided a path reference for promoting the coordinated development of rural multifunction in the traditional agricultural areas at the county scale.

## Figures and Tables

**Table 1:** The evaluation index system of rural multifunction.

Function	Index	Nature
Production function	Per capita grain output	+
	Crop output per unit area	+
	Agricultural output value per unit area	+
	Proportion of total output value of agriculture, forestry, animal husbandry and fishery	+
	Non-agricultural employment rate of rural employees	+
Life function	Engel coefficient	−
	Rural per capita electricity consumption	+
	Number of beds per 10,000 people	+
	Rural per capita net income	+
	Urban–rural income ratio	+
Ecological function	The average ecological service value	+
	Forest coverage rate	+
	Average pesticide use	−
	Average fertilizer use	−
	Average film use	−

**Table 2:** Global Moran’s I of the rural “production–life–ecological” function in Anhui.

Year	Production Function	Life Function	Ecological Function
2000	0.4532	0.3015	0.3672
2010	0.4876	0.3232	0.3883
2020	0.5036	0.3452	0.4038

**Table 3:** The Spearman correlation coefficient of rural multifunction in Anhui from 2000 to 2020.

Function Type	Production Function			Life Function			Ecological Function		
	2000	2010	2020	2000	2010	2020	2000	2010	2020
Production function	1.0000	1.0000	1.0000	0.3235 **	0.5125 **	0.5863 **	−0.1566 **	0.2821 **	−0.2235 **
Life function				1.0000	1.0000	1.0000	−0.3476 **	0.0966 **	0.1276 **
Ecological function							1.0000	1.0000	1.0000

Note: ** indicates that the correlation is significant when the confidence (double test) is 0.01.

## Data Availability

Not applicable.
